# Interleukin-6 is dispensable in pituitary normal development and homeostasis but needed for pituitary stem cell activation following local injury

**DOI:** 10.3389/fendo.2022.1092063

**Published:** 2022-12-22

**Authors:** Emma Laporte, Silke De Vriendt, Julie Hoekx, Hugo Vankelecom

**Affiliations:** Laboratory of Tissue Plasticity in Health and Disease, Cluster of Stem Cell and Developmental Biology, Department of Development and Regeneration, University of Leuven (KU Leuven), Leuven, Belgium

**Keywords:** pituitary, interleukin-6, stem cells, development, regeneration, aging

## Abstract

Recently, we discovered that the cytokine interleukin-6 (IL-6) acts as a pituitary stem cell-activating factor, both when administered *in vivo* and when added to stem cell organoid cultures *in vitro*. Moreover, its expression, predominantly localized in the gland’s stem and mesenchymal cells, promptly increases following damage in the adult pituitary, leading to stem-cell proliferative activation. Given these findings that IL-6 is involved in pituitary stem cell regulation, we addressed the question whether the cytokine has an impact on the pituitary phenotype during active phases of the gland’s remodeling, in particular embryonic development and neonatal maturation, as well as during homeostasis at adulthood and aging, all unknown today. Using the IL-6 knock-out (KO) mouse model, we show that IL-6 is dispensable for pituitary embryonic and neonatal endocrine cell development, as well as for hormonal cell homeostasis in adult and aging glands. The findings match the absence of effects on the stem cell compartment at these stages. However, using this IL-6 KO model, we found that IL-6 is needed for the acute stem-cell proliferative activation reaction upon pituitary injury. Intriguingly, regeneration still occurs which may be due to compensatory behavior by other cytokines which are upregulated in the damaged IL-6 KO pituitary, although at lower but prolonged levels, which might lead to a delayed (and less forceful) stem cell response. Taken together, our study revealed that IL-6 is dispensable for normal pituitary development and homeostasis but plays a key role in the prompt stem cell activation upon local damage, although its presence is not essentially needed for the final regenerative realization.

## Introduction

The pituitary gland is the central hub of our endocrine system, regulating key physiological processes such as body growth, metabolism, fertility, and stress. To perform this prime role, the gland accommodates multiple endocrine cell types such as somatotropes (producing growth hormone [GH]), corticotropes (adrenocorticotropic hormone [ACTH]), lactotropes (prolactin [PRL]), gonadotropes (luteinizing hormone [LH] and/or follicle-stimulating hormone [FSH]), and thyrotropes (thyroid-stimulating hormone [TSH]), as well as a population of stem cells (1–4). Upon local damage in the adult pituitary through transgenic endocrine (somatotrope) cell ablation, these resident SOX2^+^ stem cells display an acute surge in proliferative activity, followed by significant restoration of the obliterated somatotrope cells in the following 4-6 months (5, 6). Interestingly, the cytokine interleukin-6 (IL-6) was found to acutely rise in expression upon this damage infliction, predominantly occurring in the stem and mesenchymal cell populations (7). In older work, expression of IL-6 in the pituitary was reported in the folliculostellate cell population (8, 9), a heterogeneous cell compartment shown to encompass stem and mesenchymal cells (7, 10). We recently identified IL-6 as a pituitary stem cell-activating factor (7). IL-6 administration *in vivo* results in higher proliferative activity of the pituitary SOX2^+^ stem cells, while *in vitro* supplementation of IL-6 to organoid models that are derived from, and representative for, the pituitary stem cell population (11, 12), substantially increases the stem cells’ self-renewal capacity and expandability (7). Although these findings clearly show that IL-6 is involved in pituitary stem cell regulation, it is not known whether the cytokine is important in specific phases of active pituitary remodeling such as embryonic development and neonatal maturation, or in pituitary homeostasis at adulthood and aging (13). Moreover, in several other tissues such as muscle (14), liver (15) and intestine (16), IL-6 is needed for their eventual regeneration following injury. It is not known whether IL-6 occupies a similar key position in the pituitary’s regenerative realization upon damage.

To answer these questions, we applied the IL-6 knock-out (KO) mouse model (17) and phenotyped pre- and postnatal pituitary at several stages, as well as acute stem cell and regenerative responses to local injury. We found that IL-6 is expendable for normal pituitary development and homeostasis but a key mediator of the acute stem-cell activation response following local wounding. Regeneration still occurs in the absence of IL-6 which may be due to redundancy by other cytokines. This study further expands our insights in pituitary development, adult/aging homeostasis as well as regeneration, in the end needed for boosting pituitary repair in conditions of local physical damage such as trauma and tumorigenesis, or for counteracting functional decline at aging.

## Materials and methods

### Mice and *in vivo* treatment

C57BL/6 mice were used, maintained in the Animal Housing Facility of the KU Leuven under conditions of constant temperature, humidity and 12-hour light-dark cycle, with *ad libitum* access to water and food. Animal experiments were approved by the KU Leuven Ethical Committee for Animal Experimentation (P153/2018).

Mice heterozygous for the IL-6 mutation (*Il6^-/+^* (*Il6^tm1Kopf^*)), a neo^r^ cassette insertion in the first coding exon (17), were crossed to obtain homozygous offspring (*Il6^+/+^* or *Il6^-/-^*) which were analyzed at embryonic stages E12.5 and E16.5 (E0.5 as day of vaginal plug), and neonatal [postnatal day (PD) 7], (young-)adult [8-12 weeks (wks)] or middle-aged [10-15 months (mo)] phases of life. Offspring was genotyped for the presence of the neo^r^ cassette by PCR using 5’-TTCCATCCAGTTGCCTTCTTGG-3’ as common forward primer, 5’-TTCTCATTTCCACGATTTCCCAG-3’ as wildtype (WT) reverse primer and 5’-CCGGAGAACCTGCGTGCAATCC-3’ as mutant reverse primer.

*Gh^Cre/+^ (Tg(Gh1-cre)bKnmn)* mice were crossed with *ROSA26^iDTR/iDTR^* mice (*Gt(ROSA)26Sor^tm1(HBEGF)Awai^*) to create *Gh^Cre/+^;ROSA26^iDTR/+^* offspring (5), which was genotyped for the presence of the *Cre* transgene by PCR using 5’-TGCCACGACCAAGTGACAGCAATG-3’ as forward and 5’-ACCAGAGACGGAAATCCATCGCTC-3’ as reverse primer. These mice were further crossed to the *Il6^-/-^* genotype to create triple transgenic offspring, i.e.

*Gh^Cre/+^;ROSA26^iDTR/+^;Il6^-/-^* and associated genotypes *Gh^Cre/+^;ROSA26^iDTR/+^;Il6^+/+^*, *Gh^+/+^;ROSA26^iDTR/+^;Il6^+/+^* and *Gh^+/+^;ROSA26^iDTR/+^/Il6^-/-^*. The mice were intraperitoneally (i.p.) injected with diphtheria toxin (DT, 4 ng/g body weight; Sigma-Aldrich) twice a day for 3 consecutive days. Pituitaries, more in particular the major endocrine anterior lobes (ALs), were isolated and analyzed the day after DT injection (day (d) 4), one week later (d11) or 5 mo later (5).

### Embryo and pituitary isolation and dissociation

Pregnant mice were euthanized using CO_2_ asphyxiation followed by cervical dislocation. Individual embryos were isolated from the uterus and extra-embryonic membranes removed.

For isolation of the postnatal pituitary, mice were euthanized using CO_2_ asphyxiation followed by decapitation. The intact pituitary was isolated for immunostaining analysis, while the AL was separated from the intermediate lobe (InL) and posterior lobe (PL) under the stereomicroscope for dissociation into single cells with trypsin as previously described (12), to be followed by gene expression and quantitative immunofluorescence analyses.

### Immunostaining analysis

Embryos and intact pituitaries were fixed with 4% paraformaldehyde (PFA, 4% in PBS; Merck) for 24 h at 4°C and 1-3 h at room temperature (RT), respectively. Samples were dehydrated with the Excelsior ES Tissue Processor (Thermo Fisher Scientific) and embedded in paraffin using the Histostar Embedding Workstation (Thermo Fisher Scientific). Paraffin sections (5 µm), in sagittal plane for embryos and coronal plane for pituitary samples, were made with the Microm HM360 (Thermo Fisher Scientific). Sections were dewaxed, rehydrated and subjected to antigen retrieval using citrate buffer (pH 6; 30 min at 95°C). Samples were permeabilized with 0.1% Triton X-100 (in PBS; Sigma-Aldrich), incubated with blocking buffer (0.15% glycine (VWR chemicals BDH), 2 mg/mL BSA (Serva), 0.1% Triton X-100 in PBS) with 10% donkey serum (Sigma-Aldrich) for 1 h at RT, and then with primary antibodies (**Supplementary Table 1**) overnight at 4°C. Secondary antibodies (**Supplementary Table 1**) and nuclear dye Hoechst33342 (2 μg/mL; Sigma-Aldrich) were added for 1 h at RT. Sections were mounted with ProLong Gold (Thermo Fisher Scientific) and images acquired using a Leica DM5500 upright epifluorescence microscope (Leica).

For quantitative immunofluorescence analysis, dissociated AL cells were spun down onto SuperFrost glass slides (Thermo Fisher Scientific) using the Shandon CytoSpin 3 Cytocentrifuge (20,000-50,000 cells/slide; 800 rpm; 10 min). Cytospin samples were subsequently dried for 10 min, followed by a 15 min fixation in 4% PFA at RT. Cells were permeabilized with saponin (0.25% in PBS; Sigma-Aldrich) for 15 min and then blocked with donkey serum (10% in 0.25% saponin/PBS) for 20 min, both steps performed at RT. Primary antibodies were added overnight at 4°C (**Supplementary Table 1**). Cells were labelled with secondary antibodies (**Supplementary Table 1**) and nuclear dye Hoechst33342 for 1.5 h at RT, and sections mounted with ProLong Gold. Pictures were taken with a Leica DM5500 upright epifluorescence microscope. Ratios of immunoreactive cells were quantified in at least 10 random fields per slide. A range of 300-1500 cells was counted per slide using the cell counter plugin in Fiji software (18), with 2-4 slides analyzed per condition for each biological replicate. Considering the significant reduction in total cell number in the damaged AL, as found at final ablation (d11), we calculated the absolute number of the different immune-positive cells for comparisons at d11, using their proportion and the total number of cells obtained per AL, as described before (5).

### Gene expression analysis

Total RNA of embryonic pituitary, and of neonatal and adult dissociated AL cells was isolated using the RNeasy Micro kit (Qiagen) and subjected to reverse transcription (RT) with Superscript III First-Strand Synthesis Supermix (Invitrogen) according to the manufacturers’ protocol. SYBR Green-based RT-quantitative PCR (RT-qPCR) was applied on the cDNA samples using the StepOnePlus Real-Time PCR System (AB Applied Biosystems) and the Platinum SYBR Green qPCR Supermix-UDG (Thermo Fisher Scientific). Forward and reverse primers (**Supplementary Table 2**) were designed with PrimerBank (19) and PrimerBLAST (20). β-actin (*Actb*) was used as housekeeping gene for normalization, and each sample was run in duplicate. Relative gene expression levels were calculated as ΔCt values (Ct_target_ – Ct_housekeeping gene_). Gene expression levels were compared between sample and reference as relative expression ratio (fold change), calculated by the formula 2^-(ΔCt,sample – ΔCt,reference)^, or as log_2_ transformation of the fold change.

### Statistical analysis

Statistical analysis was performed (when n ≥ 3) using Graphpad Prism (v9.4.1; GraphPad Software). Statistical significance was defined as P < 0.05.

## Results

### Pituitary embryonic development is not affected by the absence of IL-6

**Figure 1:**
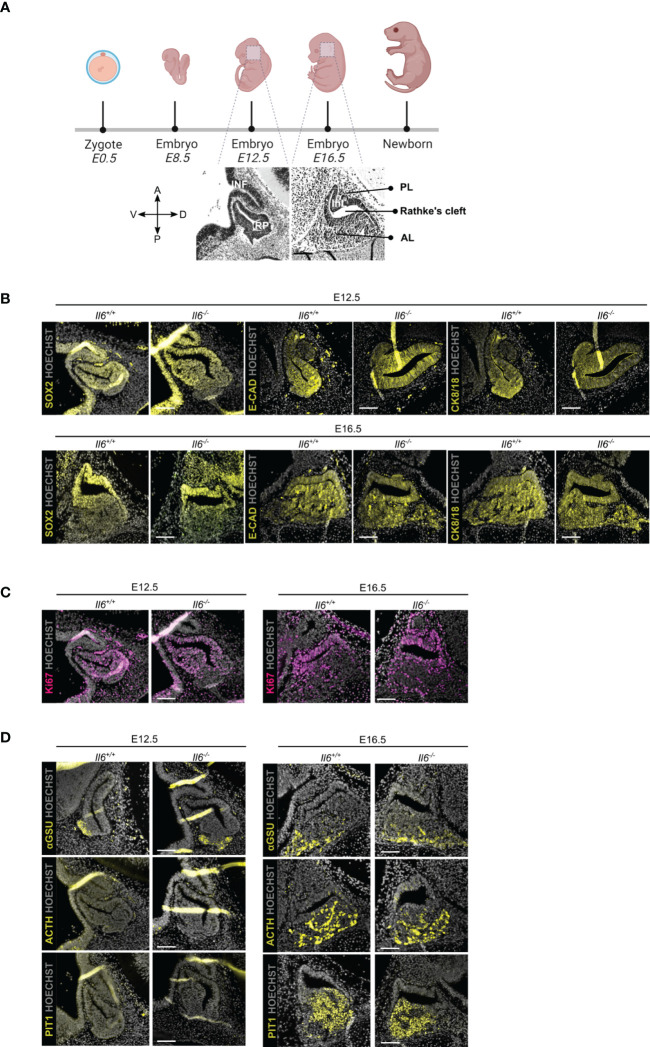
Pituitary embryonic phenotype is not affected by absence of IL-6. Overview of mouse embryonic development; embryonic day (E) 12.5 and 16.5 are highlighted and morphology of developing pituitary is shown (created with BioRender.com) **(A)**. Immunofluorescence analysis of *Il6^+/+^* and *Il6^-/-^* pituitary at E12.5 and E16.5 for SOX2, E-CAD and CK8/18 (all yellow) **(B)**, for Ki67 (magenta) **(C)** and for αGSU, ACTH and PIT1 (all yellow) **(D)**. Hoechst33342 was used as nuclear stain (grey). Scale bar, 100 μm. A, anterior; D, dorsal; P, posterior; V, ventral; INF, infundibulum; RP, Rathke’s pouch; InL, intermediate lobe; PL, posterior lobe; AL, anterior lobe.

We assessed the embryonically developing pituitary in IL-6 KO (*Il6^-/-^*) as compared to WT (*Il6^+/+^*) mice at two time points, i.e. E12.5 when the pituitary primordium Rathke’s pouch (RP) is definitively formed, and E16.5 when the different endocrine lineages [corticotrope (ACTH+), gonadotrope [glycoprotein a-subunit (aGSU)+], and somatotrope/lactotrope/thyrotrope (PIT1+)] have emerged (**Figure 1A**) (21). Of note, *Il6* is expressed in the embryonic pituitary, although at lower levels than in the adult gland (**Supplementary Figure 1**). The morphology of the developing pituitary appeared not different between *Il6^+/+^* and *Il6^-/-^* embryos (**Figures 1B-D**). In addition, the embryonic stem/progenitor cells, located around RP lumen (cleft) and marked by SOX2, as well as by the other pituitary stem cell markers E-cadherin (E-CAD) and cytokeratin (CK) 8 and 18 (11, 22), did also not display visible differences (**Figure 1B**). Similarly, the proliferative cell landscape, as visualized using the proliferation marker Ki67 and being most pronounced in the stem/progenitor cell zone around RP cleft, remained comparable between the *Il6^-/-^* and *Il6^+/+^* developing gland (**Figure 1C**). Finally, the lack of IL-6 did not discernibly affect the emergence and development of the endocrine lineages (αGSU^+^, ACTH^+^, PIT1^+^) (**Figure 1D**).

Together, IL-6 does not play an indispensable or decisive role in pituitary organogenesis, a stage during which embryonic stem/progenitor cells actively give rise to the emerging endocrine cells.

### Pituitary neonatal maturation is not affected by the absence of IL-6

**Figure 2:**
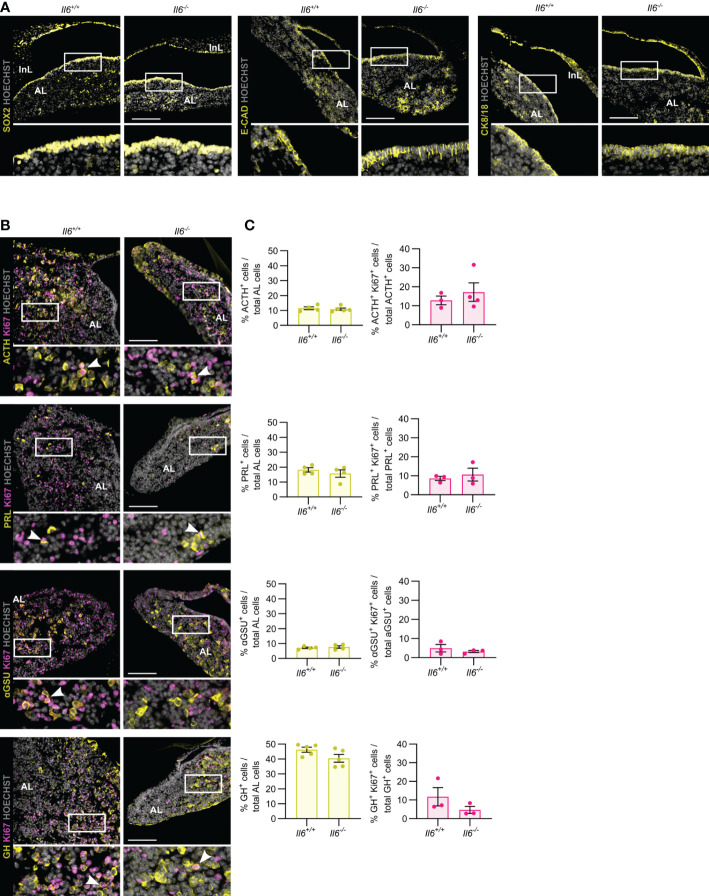
Pituitary neonatal phenotype is not affected by absence of IL-6. Immunofluorescence analysis of neonatal (PD7) *Il6^+/+^* and *Il6^-/-^* pituitary for SOX2, E-CAD and CK8/18 (all yellow) **(A)** and for ACTH, PRL, αGSU and GH (all yellow), together with Ki67 (magenta) **(B)**. Hoechst33342 was used as nuclear stain (grey). Boxed areas are magnified in the respective bottom panels. Arrowheads indicate selected double-positive cells. Scale bar, 100 μm. Proportion of hormone^+^ cells in total AL cell population (left) or of proliferating (Ki67^+^) hormone^+^ cells in the specific hormone^+^ cell compartment (right). Bars depict mean ± SEM (n = 3-5, all individually shown; unpaired t-test) **(C)**.

In the first weeks after birth, the mouse pituitary undergoes a dynamic growth and maturation process while containing an activated stem cell population (10, 13, 23–25). Here, we investigated whether IL-6 is involved in this active neonatal pituitary remodeling. *Il6* is expressed in the neonatal (PD7) gland, but still lower than in the later adult stage (**Supplementary Figure 1**) (10). The stem cell compartment (encompassing the marginal zone lining the residual RP cleft), as marked by SOX2, E-CAD and CK8/18, did not show overt differences between *Il6^+/+^* and *Il6^-/-^* neonatal pituitary (**Figure 2A**), in line with our recent findings that the number of SOX2^+^ cells and their proliferating subfraction does not change in IL-6 KO neonatal pituitary (10). Similarly, the topography and size of the different endocrine cell populations (i.e. ACTH^+^, PRL^+^, αGSU^+^, GH^+^) was not affected in the *Il6*-deficient neonatal pituitary (**Figures 2B, C**), robustly substantiating and expanding our recent findings of unaffected gene expression of lineage progenitor and endocrine cell markers (10). In addition, proliferation within the individual hormonal cell lineages did also not significantly change in *Il6^-/-^ versus Il6^+/+^* condition (**Figures 2B, C**).

Taken together, IL-6 is found to be dispensable for the pituitary’s neonatal maturation process encompassing activated stem cells and expanding endocrine lineages.

### Pituitary adult homeostasis and aging phenotype are not affected by the absence of IL-6

**Figure 3:**
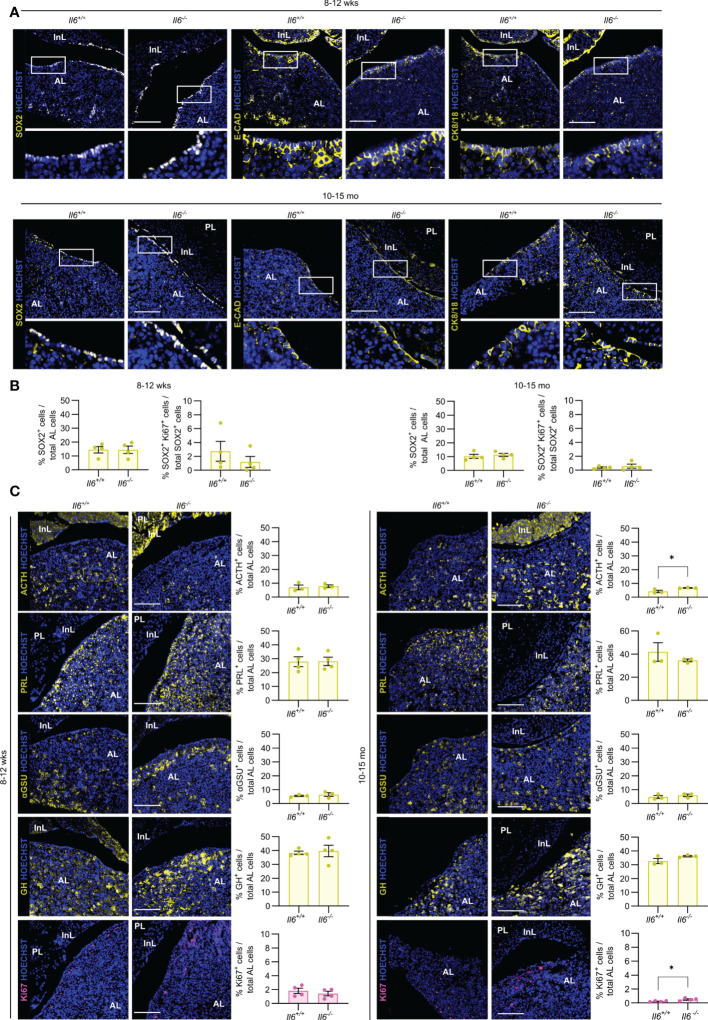
Pituitary adult and aging phenotypes are not affected by absence of IL-6. Immunofluorescence analysis of adult (8-12 wks) and aging (10-15 mo) *Il6^+/+^* and *Il6^-/-^* pituitary for SOX2, E-CAD and CK8/18 (all yellow). Hoechst33342 was used as nuclear stain (blue). Boxed areas are magnified in the respective bottom panels. Scale bar, 100 μm **(A)**. Proportion of SOX2^+^ cells in total AL cell population or of proliferating SOX2^+^Ki67^+^ cells in the SOX2^+^ cell compartment. Bars depict mean ± SEM (n = 4, all individually shown; unpaired t-test) **(B)**. Immunofluorescence analysis of adult and aging *Il6^+/+^* and *Il6^-/-^* pituitary for ACTH, PRL, αGSU, GH (all yellow) and Ki67 (magenta). Hoechst33342 was used as nuclear stain (blue). Scale bar, 100 μm (left). Proportion of hormone^+^ or Ki67^+^ cells in total AL cell population (right). Bars depict mean ± SEM (n = 3-4, all individually shown; unpaired t-test, *P < 0.05.) **(C)**.

Then, we assessed the impact of IL-6 absence on the pituitary stem and endocrine cell phenotype of (young-)adult (8-12 weeks-old) and aging (10-15 months-old) mice, in which *Il6* is prominently expressed [(7) and **Supplementary Figure 1**]. Lack of IL-6 did not visibly impact the expression pattern of the stem cell markers SOX2, E-CAD and CK8/18 in both adult and aging gland (**Figure 3A**), neither is there an overt (significant) change in the proportion of SOX2^+^ cells or their proliferative subfraction (**Figure 3B**), findings that further expand and corroborate our recent observations as analyzed for adult mice (7). Furthermore, absence of IL-6 did generally not entail significant changes in the endocrine cell lineages or proliferating (Ki67^+^) cells, except for a small increase in corticotropes (ACTH^+^ cells) and in proliferative cells in the aging *Il6^-/-^* pituitary (**Figure 3C**), of which the (biological) relevance is presently unclear (see Discussion).

Taken together, IL-6 is not essential in the homeostatic process of adult and aging pituitary, during which cell turnover is very limited and stem cells are highly quiescent (2, 3, 7, 13).

### IL-6 is needed for the acute stem cell activation upon local injury, but not for the eventual regeneration

**Figure 4:**
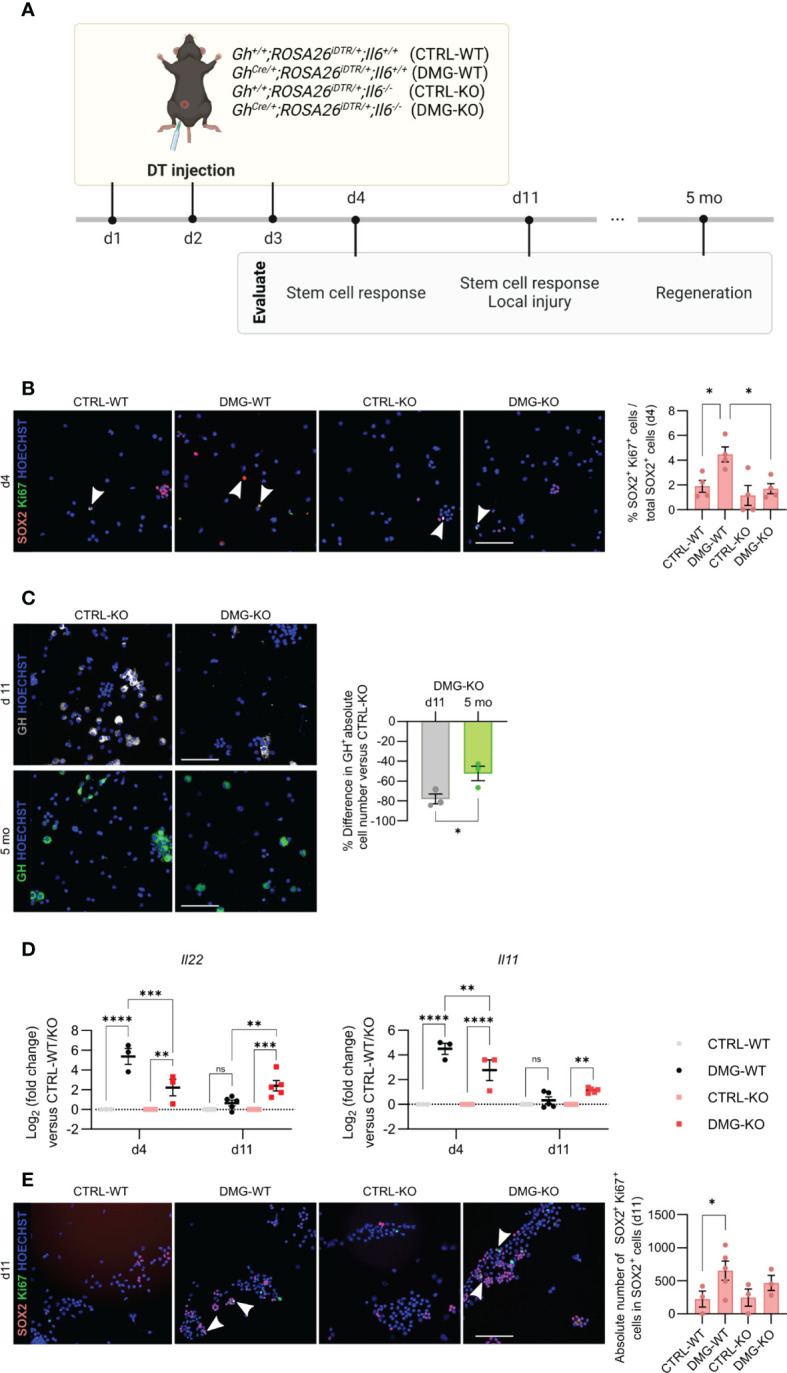
IL-6 is necessary for the acute stem cell activation upon local injury, but dispensable for the eventual regeneration. Overview of the experimental set-up (created with BioRender.com) **(A)**. Immunofluorescence analysis of AL cell cytospin samples from CTRL-WT, DMG-WT, CTRL-KO and DMG-KO mice for SOX2 (magenta) and Ki67 (green) at d4. Hoechst33342 was used as nuclear stain (blue). Arrowheads indicate double-positive cells. Scale bar, 100 μm (left). Proportion of proliferating SOX2^+^Ki67^+^ cells in the SOX2^+^ cell compartment (right). Bars depict mean ± SEM (n = 4, all individually shown; one-way ANOVA with Tukey’s multiple comparisons test, *P < 0.05) **(B)**. Immunofluorescence analysis of AL cell cytospin samples of CTRL-KO and DMG-KO mice for GH (grey or green) at d11 and 5 mo. Hoechst33342 was used as nuclear stain (blue). Scale bar, 100 μm (left). Percent difference in pituitary absolute GH^+^ cell number compared to CTRL-KO at d11 and 5 mo (right). Bars depict mean ± SEM (n = 3, all individually shown; unpaired t-test, *P < 0.05) **(C)**. Gene expression levels of *Il22* and *Il11* in DMG-WT and DMG-KO AL at d4 and d11, relative to CTRL-WT and CTRL-KO (dotted line), respectively. Graphs show mean ± SEM (n = 3-5, all individually shown; two-way ANOVA with Tukey’s multiple comparisons test, **P < 0.01, ***P<0.001, ****P<0.0001; ns, non-significant) **(D)**. Immunofluorescence analysis of AL cell cytospin samples of CTRL-WT, DMG-WT, CTRL-KO and DMG-KO mice for SOX2 (magenta) and Ki67 (green) at d11. Hoechst33342 was used as nuclear stain (blue). Arrowheads indicate double-positive cells. Scale bar, 100 μm (left). Proportion of proliferating SOX2^+^Ki67^+^ cells in the SOX2^+^ cell compartment (right). Bars depict mean ± SEM (n = 3-4, all individually shown; paired t-test, *P < 0.05) **(E)**. DT, diphtheria toxin.

Following local injury in the adult pituitary, as inflicted by somatotrope cell ablation using the *Gh^Cre/+^;ROSA26^iDTR/+^* mouse model, the quiescent stem cells become promptly activated and enhance in proliferative activity (5–7, 26). Here, damage was inflicted in triple transgenic *Gh^Cre/+^;ROSA26^iDTR/+^;Il6^-/-^* mice (further referred to as damaged (DMG)–KO) by DT injection for 3 consecutive days, and the pituitary compared to the appropriate DT-injected controls [*Gh^+/+^;ROSA26^iDTR/+^;Il6^+/+^* (CTRL-WT), *Gh^Cre/+^;ROSA26^iDTR/+^;Il6^+/+^* (DMG-WT), *Gh^+/+^;ROSA26^iDTR/+^;Il6^-/-^* (CTRL-KO)] (**Figure 4A**). We observed a complete absence of the acute (d4) proliferative stem cell reaction (i.e. increase in SOX2^+^/Ki67^+^ cells as found in DMG-WT) when IL-6 is lacking (**Figure 4B**). This finding is in line with, but much more pronounced than, the 50% reduction in proliferative SOX2^+^ cell response that we recently reported through pharmacological IL-6 inhibition by *in vivo* antibody administration (7).

In previous work, we discovered that the adult pituitary possesses regenerative competence upon injury, as demonstrated by substantial (~50%) regeneration of the somatotrope cell population, observed 4-6 months after their obliteration (5, 6). Intriguingly, despite the lack of an acute stem cell reaction in the absence of IL-6, we here still detected significant somatotrope regeneration levels (~40%) in the DMG-KO pituitary when analyzed 5 months after the somatotrope ablation (**Figures 4A, C**), reaching a GH^+^ cell proportion similar to that in DMG-WT gland (**Supplementary Figure 2A**), thereby indicating comparable regeneration grade. The finding of regenerative realization is not due to lower extent of damage (ablation grade) inflicted in DMG-KO pituitary, which is indeed comparable (~80%; **Figure 4C**) to the grade as reported before in the presence of IL-6 (5, 6). As another possible explanation, we examined whether other cytokines of the IL-6 family, or related factors, may rescue the function of IL-6 after pituitary damage in the IL-6 KO mouse. Expression levels of these cytokines in basal undamaged pituitary were not different between *Il6^+/+^* and *Il6^-/-^* AL, except for *Il22* which was lower in the IL-6 KO AL (**Supplementary Figure 2B**). Interestingly, the expression of *Il22*, as well as of *Il11*, substantially increased immediately after pituitary damage (d4) in the IL-6 WT animals, which quickly returned to baseline levels one week later (d11; DMG-WT in **Figure 4D**). Of note, expression of IL-6, also upregulated immediately after damage, remained higher at d11 (**Supplementary Figure 2C**). *Il22* and *Il11* expression was also increased in the absence of IL-6 (DMG-KO; **Figure 4D**) although at a significantly lower level than in DMG-WT (**Figure 4D**). Intriguingly, levels did not return to control one week later but remained elevated (**Figure 4D**). A similar pattern of sustained expression, different from control, was found for *Lif* but not for the other cytokines tested (*Cntf, Osm*, *Ifng*) (**Supplementary Figure 2D**). Finally, the number of SOX2^+^Ki67^+^ cells in the pituitary stem cell compartment showed a slight trend of increase, although not significant, in DMG-KO *versus* CTRL-KO pituitary at this later timepoint (d11; **Figure 4E**). Together, these findings advance the hypothesis that, in the absence of IL-6, the lower but prolonged upregulation of other cytokines may compensate for the lack of IL-6 and might still generate a stem cell reaction although in a delayed and less forceful manner. However, it should be noted that this interpretation is still hypothetical and should be taken with the necessary caution (see Discussion).

**Figure 5:**
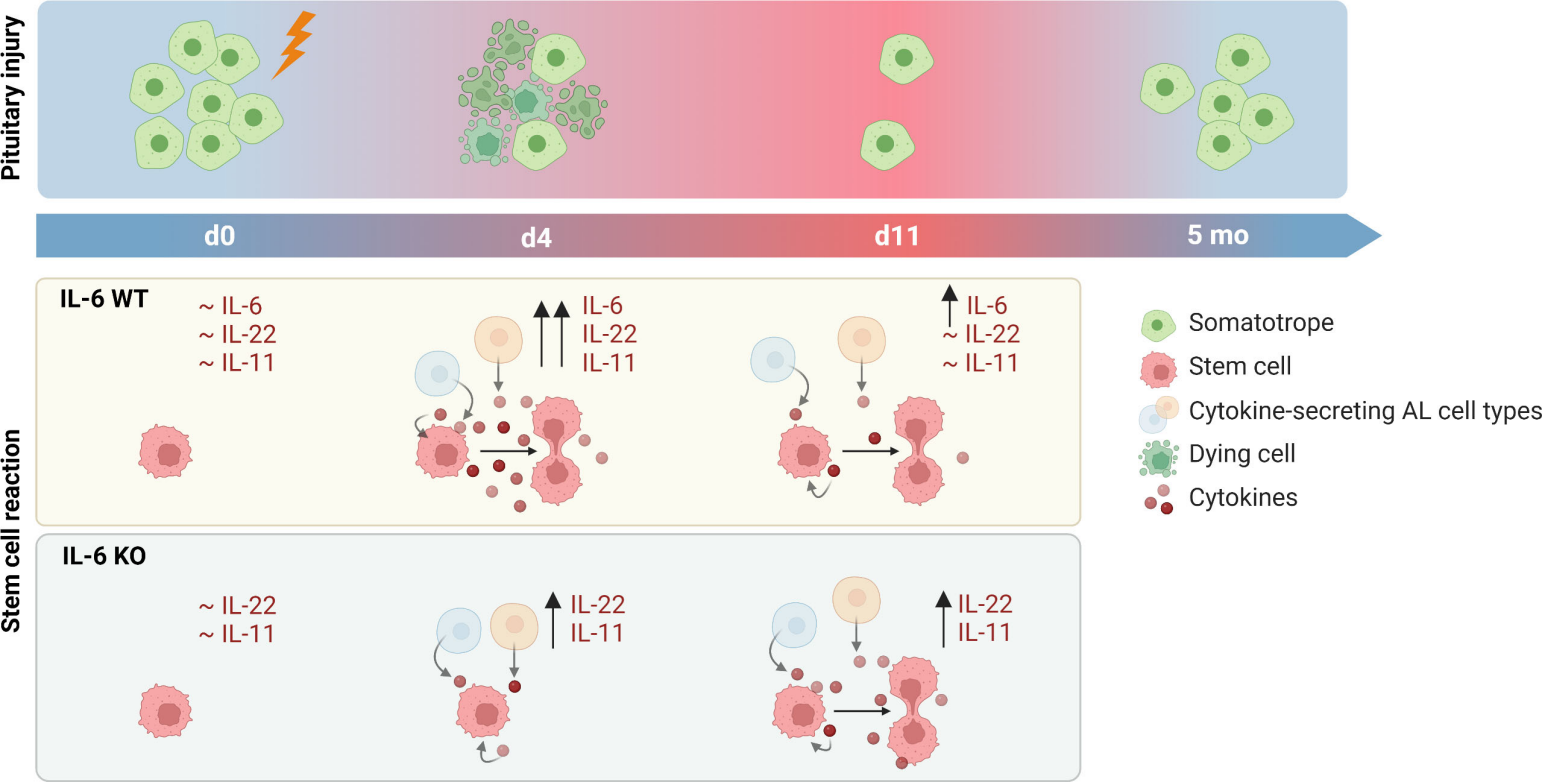
Proposed hypothesis on role of IL-6 in pituitary stem cell activation and regeneration upon local injury. In unperturbed (IL-6 WT) conditions, levels of cytokines are at basal (low) level. Upon local damage, expression is promptly upregulated (d4) and stem cells are activated into proliferation. One week later (d11), levels of cytokines except IL-6 have returned to basal levels. In the absence of IL-6 (IL-6 KO), cytokines (in particular IL-22 and IL-11) are also acutely upregulated (d4) following local injury although at lower level than in IL-6 WT conditions, while stem cells do not show activation at this timepoint. However, expression levels remain high for a longer period (d11), possibly resulting in stem-cell proliferative activation which thus might occur in a delayed manner in the absence of IL-6. In both IL-6 WT and KO conditions, significant regeneration of the ablated endocrine cells is eventually realized (created with BioRender.com).

In conclusion, IL-6 plays a key role in the acute pituitary stem cell activation response upon local damage. In its absence, other cytokines such as IL-11 and IL-22 may compensate, with lower but prolonged expression, thereby potentially entailing a delayed (and less potent) stem cell activation response toward eventual regenerative realization (summarized as hypothetical model in **Figure 5**).

## Discussion

In the present study, we explored the pituitary’s phenotype in the absence of IL-6 using the IL-6 KO mouse model to assess its importance in embryonic development, neonatal maturation, homeostasis at adulthood and aging, and stem cell reaction and regeneration upon local injury.

In embryonic and neonatal development, IL-6 deficiency does not affect pituitary morphology nor stem and endocrine cell phenotypes. These findings may be in line with only low expression of *Il6* at these early ages. In addition, or alternatively, compensation by other cytokines may occur as we recently showed that *Il11*, *Lif* and *Tnf* are increased in the neonatal *Il6^-/-^* pituitary (10). Compensation of IL-6 absence by other cytokines has also been reported in other tissues. For example, upregulation of TNFα functionally compensates for the lack of IL-6 in induced systemic inflammation (27). Redundancy of cytokines signaling through the common gp130 co-receptor (such as IL-6, IL-11 and LIF) has also been shown before. For instance, in adult hematopoiesis IL-11 deficiency is compensated for by IL-6 or LIF (28). Along the same line, although mice lacking individual members of the IL-6 family display only mild phenotypes, animals lacking gp130 are not viable (29). Thus, specific conditional knock-out of gp130 in the pituitary (stem and/or endocrine) cells would be needed to discern gp130-associated cytokine redundancy in the developing pituitary (30).

In the adult gland, *Il6* expression is higher and found to be predominantly expressed by the stem and mesenchymal cell populations (7). Yet, in unperturbed homeostatic conditions, no changes in stem and endocrine cell phenotypes were observed in the IL-6-deficient gland. These findings are considered to be in line with the overall slow turnover (31) and highly quiescent nature of the stem cells in the adult gland (2, 3, 7, 13). Indeed, a role for IL-6 in crypt homeostasis has been observed in the intestine which is a highly dynamic tissue with very active stem cells (32). Nevertheless, upon local pituitary injury, the resident stem cells become rapidly driven into proliferative behavior (5–7). This prompt stem cell activation reaction is not observed in the *Il6^-/-^* pituitary. Similar findings were reported in the intestine in which lower crypt proliferation was observed in IL-6 KO mice upon local epithelial wounding (16). Thus, IL-6 plays a key role in the prompt pituitary stem cell reaction to local damage. However, less expectedly, the ensuing regeneration still occurs in the absence of IL-6. Other cytokines, in particular *Il22* and *Il11*, were also found to be upregulated upon injury. Although occurring at lower level in the IL-6 KO mouse, the cytokines remained elevated for a longer period, which might entail a delayed (and less forceful) stem cell-proliferative reaction in the absence of IL-6 (**Figure 5**). Although this interpretation should still be taken with the necessary caution, it is interesting to see that a similar observation was reported in skeletal muscle injury, in which the initial reaction was delayed in *Il6^-/-^* mice, but full recovery of the muscle mass was still achieved, leading to the authors’ conclusion that “IL-6-regulated processes occurring early in the recovery process may affect the initial recovery rate, but are not required if sufficient recovery time is allowed” (33).

Finally, we here corroborated our previous observations in the aging pituitary (6, 7) of decline in number and functional (proliferative) activity of SOX2^+^ stem cells when compared to young-adult gland (compare age panels in **Figure 3B**). In addition, we found that proliferating (Ki67^+^) cells are increased, although subtly, in the IL-6 KO pituitary. We have previously discovered that the aging pituitary suffers from inflammaging [referring to chronic low-grade inflammation that develops with advancing age (34)], typified by a pronounced inflammatory phenotype including high levels of IL-6 (7). Absence of IL-6 may, at least partly, relieve this restrictive impact of the inflammatory milieu on pituitary cell behavior (such as proliferation, being at low level). A trend of higher proliferation is also seen specifically in the stem cell population although not statistically significant (**Figure 3B**, right panels, 0.37% in *Il6^+/+^ versus* 0.58% in *Il6^-/-^*), which is in line with the regained functionality of aging pituitary stem cells when cultured as organoids outside of the inflammatory obstruction (7). Whether the increased proliferation of corticotrope (ACTH^+^) cells should also be considered in this context, is not clear.

In conclusion, our study shows that IL-6 is dispensable for normal pituitary early-development and homeostasis. However, upon perturbation by local tissue injury, IL-6 is needed for a prompt and maximal stem cell activation. These findings expand our knowledge on pituitary (stem cell) regulation which in the future may help in translational efforts toward restoring and rejuvenating pituitary function.

## Data availability statement

The original contributions presented in the study are included in the article/**Supplementary Material**. Further inquiries can be directed to the corresponding author.

## Ethics statement

The animal study was reviewed and approved by KU Leuven Ethical Committee for Animal Experimentation (P153/2018).

## Author contributions

EL and SV designed the concepts and experiments, performed the experiments and the data analysis, interpreted the results and co-wrote the manuscript; JH helped in executing experiments; HV supervised the entire project, co-developed the concepts and ideas, co-designed the experiments, co-interpreted the data and co-wrote the manuscript. All authors contributed to the article and approved the submitted version.
