# Supplementary figures and images for: Interleukin-6 is dispensable in pituitary normal development and homeostasis but needed for pituitary stem cell activation following local injury
Source: Front Endocrinol (Lausanne). 2022 Dec 22;13:1092063. doi: 10.3389/fendo.2022.1092063 (PMC9815540; doi:10.3389/fendo.2022.1092063)

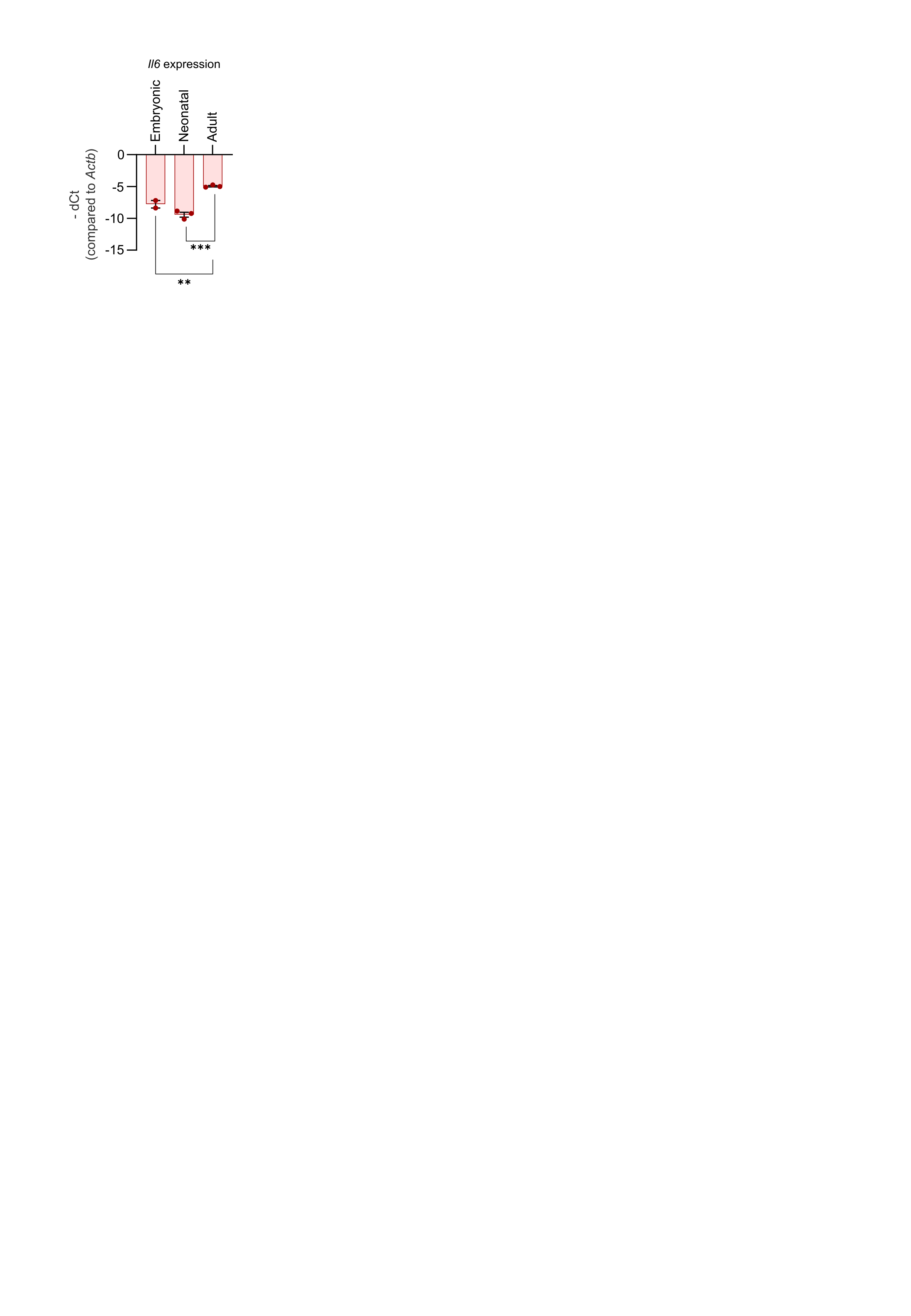

Supplement: Supplementary file 2 [file Image_1.tif]

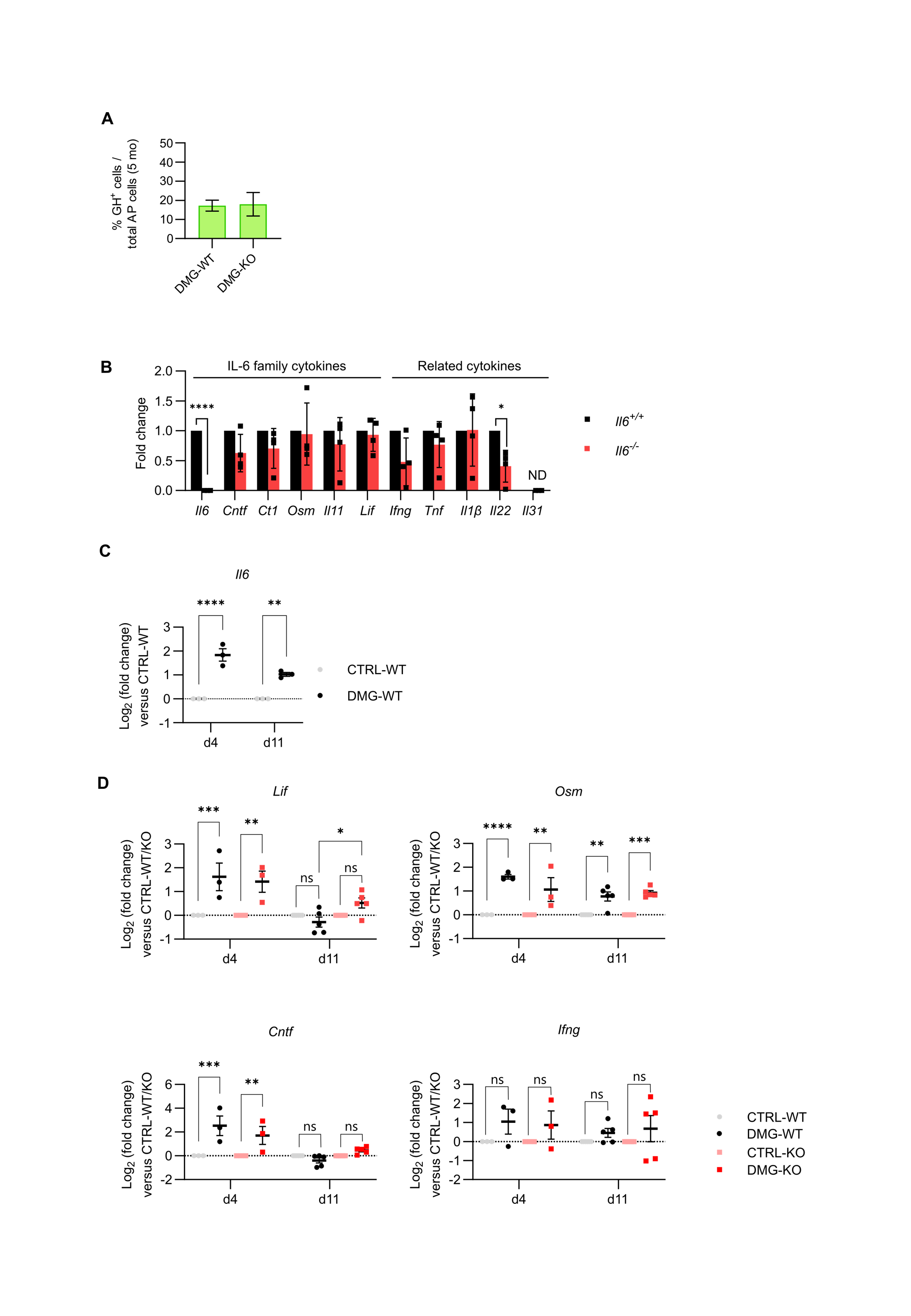

Supplement: Supplementary file 3 [file Image_2.tif]
